# Neuropsychiatric Profile as a Predictor of Cognitive Decline in Mild Cognitive Impairment

**DOI:** 10.3389/fnagi.2021.718949

**Published:** 2021-12-08

**Authors:** Natalia Roberto, Maria J. Portella, Marta Marquié, Montserrat Alegret, Isabel Hernández, Ana Mauleón, Maitee Rosende-Roca, Carla Abdelnour, Ester Esteban de Antonio, Juan P. Tartari, Liliana Vargas, Rogelio López-Cuevas, Urszula Bojaryn, Ana Espinosa, Gemma Ortega, Alba Pérez-Cordón, Ángela Sanabria, Adelina Orellana, Itziar de Rojas, Sonia Moreno-Grau, Laura Montrreal, Emilio Alarcón-Martín, Agustín Ruíz, Lluís Tárraga, Mercè Boada, Sergi Valero

**Affiliations:** ^1^Ace Alzheimer Center Barcelona, Universitat Internacional de Catalunya (UIC), Barcelona, Spain; ^2^Department of Psychiatry and Forensic Medicine, Universitat Autònoma de Barcelona (UAB), Barcelona, Spain; ^3^Department of Psychiatry, Institut d’Investigació Biomèdica Sant Pau (IIB Sant Pau), Hospital de la Santa Creu i Sant Pau, Barcelona, Spain; ^4^Networking Research Center on Mental Health (CIBERSAM), Madrid, Spain; ^5^Networking Research Center on Neurodegenerative Diseases (CIBERNED), Madrid, Spain

**Keywords:** mild cognitive impairment, cognitive decline, neuropsychiatric symptoms, irritability, apathy, anxiety, depression

## Abstract

**Introduction:** Mild cognitive impairment is often associated with affective and other neuropsychiatric symptoms (NPS). This co-occurrence might have a relevant impact on disease progression, from MCI to dementia.

**Objective:** The aim of this study was to explore the trajectories of cognitive decline in an MCI sample from a memory clinic, taking into consideration a perspective of isolated cognitive functions and based on NPS clusters, accounting for the different comorbid symptoms collected at their baseline visit.

**Methods:** A total of 2,137 MCI patients were monitored over a 2.4-year period. Four clusters of NPS (i.e., Irritability, Apathy, Anxiety/Depression and Asymptomatic) were used to run linear mixed models to explore the interaction of cluster with time on cognitive trajectories using a comprehensive neuropsychological battery (NBACE) administered at baseline and at the three subsequent follow-ups.

**Results:** A significant interaction between cluster and time in cognitive decline was found when verbal learning and cued-recall were explored (*p* = 0.002 for both memory functions). For verbal learning, the Irritability cluster had the largest effect size (0.69), whereas the Asymptomatic cluster showed the smallest effect size (0.22). For cued-recall, the Irritability cluster had the largest effect size among groups (0.64), and Anxiety/Depression had the smallest effect size (0.21).

**Conclusions:** In MCI patients, the Irritability and Apathy NPS clusters shared similar patterns of worsening in memory functioning, which could point to these NPS as risk factors of a faster cognitive decline, acting as early prognostic markers and helping in the diagnostic process.

## Introduction

Biological changes bonded to impairment of cognitive functions are shown as humans age (Glisky, 2007). In the elderly, some cognitive skills such as attention, memory, executive functions or processing speed suffer from subtle changes associated with the normal aging process (Park et al., 2002; Park and Reuter-Lorenz, 2009), whereas others suffer a greater cognitive decline beyond expected, but not all decrement in cognitive functioning in this population is a precursor of disease. Therefore it is important to distinguish between normal and pathological cognitive decline, mainly because it could affect the patient’s daily functioning (Ginsberg et al., 2019) worsening their quality of life. The accurate measurement of cognitive decline over time is of utmost importance as it could help in the diagnosis and posterior prognosis of different neurodegenerative diseases and other syndromes (Grober et al., 2008; Wise et al., 2019).

Cognitive impairment is often associated with affective symptoms, such as anxiety or depression (Geda et al., 2008; Hermida et al., 2012; Singh-Manoux et al., 2017), which have been widely reported in different populations. While more is known about the former in relation to cognitive decline (Gonzales et al., 2017), there is still not much agreement about other neuropsychiatric symptoms (NPS) that could interfere with or relate somehow to a worsening in neuropsychological measures over time in early stages of different diseases. Some studies demonstrate the co-existence of both factors, with NPS being the predecessors of cognitive decline, often for many years (Wise et al., 2019; Tsunoda et al., 2020). There is no consensus on the order of appearance of both neural insults; previously, it was thought that cognitive deficits were the main reason for medical consultation, while studies increasingly claimed that NPS were the precursors initially detected before any cognitive decline is shown (Mortby and Anstey, 2015; Ismail et al., 2018). In any event, it is important to delve into early cognitive decline and try to elucidate the factors favoring it. At this early stage, another important feature to keep in mind is that comorbid NPS are often found in the clinical practice, and this co-occurrence of NPS and cognitive decline might have a cumulative effect on disease progression (Geda et al., 2013). Many attempts have been made to identify specific profiles of NPS associated with Alzheimer’s disease (AD). Some studies have explored the existence of neuropsychiatric subsyndromes or the genetics of NPS that could be the basis of AD, but no clear conclusions have been raised so far (Canevelli et al., 2013; Huang et al., 2020). A high prevalence of NPS in AD has commonly been associated with a worsening in the patient’s functionality (Karttunen et al., 2011).

It is well known that NPS seem to play a critical role in early clinical stages of the dementia continuum (Karttunen et al., 2011; Burhanullah et al., 2020), such as in Mild Cognitive Impairment (MCI) (Lyketsos et al., 2002; Geda et al., 2008; Peters et al., 2012). In a search of profiles of clustered symptoms that could serve as markers of disease progression in early stages, NPS would act as early clinical manifestations of an emergent process of neurodegeneration (Gallagher et al., 2017). In particular, affective NPS (depression, apathy, anxiety and irritability) were associated with a more rapid progression to AD in older adults with MCI (Jang et al., 2020), and those have also even shown synergic effects with the APOE ε4-allele (Valero et al., 2020). Recently, some attempts have been made to investigate grouped NPS as possible predictors of cognitive decline along the progression of MCI toward dementia (Palmer et al., 2007; Edwards et al., 2009). In a recent 2-year prospective study, and according to the three classes found in terms of NPS trajectories (*stable, improved* and *worsened*) in MCI patients, it was found that the NPS *worsened* class suffered the greatest cognitive and functional decline, as well as the highest conversion rate in comparison with the *stable* class and the *improved* class (David et al., 2016). Other clinical studies exploring associations of NPS by using factor analysis in MCI and mild AD dementia were focused on conversion to dementia and/or its relation to the severity of cognitive decline, but not specific cognitive domains (Siafarikas et al., 2018; Liew, 2019). There are two studies in the same line exploring NPS clusters and conversion to dementia in cognitively healthy volunteers (Leoutsakos et al., 2015; Forrester et al., 2016). However, there is still no consensus in the findings, probably due to dissimilarities in the design and methodology of these studies (different diagnostic criteria, sample selection or neuropsychological assessment applied) (Ma, 2020). Likewise, there is a conceptual void when exploring the most common NPS in patients with MCI and their implications in cognitive decline in a long-term follow-up to analyze patients’ progression in specific domains. Only a few studies in neurological patients, such as those with Parkinson’s and Huntington’s diseases (Pirogovsky-Turk et al., 2017), are going in this direction of assessing NPS in the MCI population and their implications for cognitive decline (Weintraub et al., 2015; Donaghy et al., 2018).

Therefore, this longitudinal study aims at investigating the existence of different trajectories of specific cognitive-domain decline over time in an MCI sample from a memory clinic, considering baseline NPS clustering.

## Materials and Methods

### Participants

The study was conducted at the Memory Clinic of ACE Alzheimer Center Barcelona (Spain), a private non-profit institution focused on the diagnosis, care and research of cognitive disorders and providing services to the Catalan Public Health Service (Xarxa Hospitalària d’Utilització Pública, XHUP) (Boada et al., 2014).

A total of 2,137 patients diagnosed with MCI were selected from a pool of patients evaluated at the Memory Clinic, see Roberto et al. (2021) for more information; MCI subtypes diagnoses were based on modified Petersen’s criteria and Lopez and colleagues’ classification, defined as amnestic (aMCI) or non-amnestic (naMCI), and possible or probable MCI due to AD, respectively (Petersen et al., 1999; Lyketsos et al., 2002; Petersen, 2004). All patients had to fulfill the following inclusion criteria: (i) more than 44 years old; (ii) a Mini-Mental State Examination (MMSE) total score of 24 or above; (iii) a Clinical Dementia Rating (CDR) score of 0.5; (iv) a Global Deterioration Scale (GDS) score of three or below; (v) at least six total years of formal education; (vi) absence of severe visual or auditory disturbances that could hinder the neuropsychological examination; (vii) presence of an informant or relative to complete the baseline administration of the NPI-Q; and, (viii) a baseline neuropsychological visit completed along with at least one follow-up. All clinical data were collected from January 2006 to June 2017. In all cases, the date of the MCI diagnosis was taken as the starting point or inclusion date for this study. Patients were followed up approximately annually with a clinical assessment that included a neurology and a neuropsychological visit.

### Cognitive Measures

Cognitive data were collected at baseline and at every follow-up visit, using *The Neuropsychological Battery of Fundació ACE* (NBACE). The NBACE is a 50-min battery designed to assess cognitive domains especially affected in the elderly when dementia due to AD or other neurodegenerative processes is suspected (Alegret et al., 2012). The NBACE was proposed as a brief, easy-to-administer and goal-directed compilation of globally-used neuropsychological tests in our target population, provided that it is focused on verbal memory and learning, visual perception, and executive functions, which are affected early in the course of the disease. However, these are not the only explored domains. In our study we included tests sensitive to the following cognitive domains: attention, working memory, processing speed, executive functions, verbal memory, language, gnosis, visuospatial skills and praxis. Normative data and cut-off scores of the NBACE subtests for individuals more than 44 years old can be found elsewhere (Alegret et al., 2013).

Processing speed was measured with the Automatic Inhibition subtest of the *Syndrome Kurtz Test* (SKT; Erzigkeit, 1989), using execution time as the raw score. Attention and working memory scores were obtained by means of the digit span forward and backward subtests of the (WAIS–III; Wechsler, 1997a). Verbal learning and memory were measured through the word list learning test from the *Wechsler Memory Scale–Third Edition*. Verbal learning trials, long-term retention and cued-recall were used as raw scores; the interference list was not included in the battery (WMS–III; Wechsler, 1997b). Verbal learning was composed of the sum of raw scores obtained in the four trials of the learning phase (Σ1^st^ + 2^nd^ + 3^rd^ + 4^th^); long-term retention was the total amount free recalled words; and cued-recall was the total number of words correctly recognized among the correct items and the same amount of “interference” items. Language was measured with the 15-item version of the *Boston Naming Test* (BNT; Kaplan et al., 1983). Gnosis, with a single score, was evaluated by means of *the Poppelreuter test* (Della Sala et al., 1995). Visuoconstructive praxis was evaluated with the abbreviated *block design* subtest of the *Wechsler Adult Intelligence Scale–Third Edition* WAIS–III (Wechsler, 1997a). Visuospatial skills were measured with *Luria’s Clock Test* (Golden, 1980), providing a single score. Finally, executive functioning was measured through different tests: the Automatic Inhibition of the SKT accuracy score of inhibition ability; phonetic, semantic and verb fluencies, obtaining three scores derived from the number of words recalled; and the abbreviated similarities subtest of the WAIS-III for abstract reasoning.

### Neuropsychiatric Symptoms Measures

Neuropsychiatric symptoms were evaluated at the baseline clinical assessment using the Neuropsychiatric Inventory-Questionnaire (NPI-Q) (Boada et al., 2002). The NPI-Q is a simplified and widely-used scale that assesses 12 behavioral disturbances including delusions, hallucinations, agitation/aggression, depression/dysphoria, anxiety, elation/euphoria, apathy/indifference, disinhibition, irritability/lability, aberrant motor behavior, sleep and night-time behaviors, and appetite and eating disorders, in the dementia-related population. The NPI-Q was completed through information provided by a patient’s reliable informant (family member or caregiver). A change during the previous month in each one of the 12 behavioral domains was recorded as a dichotomized measure (present or absent). For more details on this measure and procedures see Roberto et al. (2021).

### Analytical Approach

The present study is based on the results of a previous Latent Class Analysis (LCA) for clustering participants by means of a dichotomized NPI-Q measure (Roberto et al., 2021). Each participant was assigned to the best fitting cluster with the highest membership probability using baseline NPS. A 4-cluster was considered the optimal solution: Class 1 = *Irritability*; Class 2 = *Apathy*; Class 3 = *Anxiety/Depression*; Class 4 = *Asymptomatic*. Then, linear mixed-effects models (LMMs) were executed to explore cognitive decline for specific domains including NPS clusters and time of assessment (four time points, baseline, 1−, 2−, and 3-year follow-ups). Individual LMM models were calculated, one for each neuropsychological domain explored. Interaction of NPS cluster by time of assessment was considered the main effect of interest in the model, also incorporating the corresponding cluster and time main effects. For these two factors, fix and also as a random effect were analyzed, considering that assessment time points could vary among participants, and they also had different conditional probabilities of cluster belonging (see Roberto et al., 2021), i.e., individual differences had to be modeled. Mean differences (SD) accounting for time between baseline and every follow-up (times of assessments) were as it follows: from baseline to the first follow up were 11.26 months (6.22); from baseline to the second follow-up were 22 months (7.48); and from baseline to the third follow-up were 32.21 months (9.01). Both random intercept and slopes were included in the analyses. Asymptomatic class was considered the reference category. As a controlling factors, age, MMSE, educational level, sex, conversion to dementia (yes/no), MCI type (amnestic/non-amnestic), and MCI profile (possible/probable) were also included in the models and were considered in the models as fixed factors. Only when a significant interaction (cluster × time) was obtained in a specific cognitive domain, simple effects were calculated contrasting differences among clusters across the time points. Syntax of LMM is provided in Supplementary Material.

## Results

According to previously published findings from our group (Roberto et al., 2021), the whole sample of 2,137 MCI patients was divided into four NPS clusters. Class 1-*Irritability* included 134 patients (6.3%) with high probability of irritability (0.93), together with lower probability of anxiety (0.64) and apathy (0.63). Class 2-*Apathy* comprised 272 patients (12.7%) and it was strongly represented by this symptom (1). Class 3-*Anxiety/Depression* included 1,056 patients (49.4%) who showed a high probability of depression (0.95), anxiety (0.93) and, by far, apathy (0.61). Class 4-*Asymptomatic* included 675 patients (31.6%) with low probabilities (<0.3) in all NPS.

Demographic and clinical characteristics of the sample stratified by NPS cluster are shown in Table 1. There were significant differences in age, gender, educational level, MMSE total score, MCI type, MCI profile, and conversion rates to dementia among the four NPS clusters, thus those were included in the models and were considered as controlling factors. Differences among groups in age distribution showed that *Apathy* patients were the oldest (mean age 76.2). In relation to gender, women were more prevalent in the *Anxiety/Depression* and *Asymptomatic* classes (65.2 and 60.9%, respectively). Educational level attained was higher in patients in the *Apathy* class (8.04 years of education). In relation to MMSE score obtained, the *Anxiety/Depression* class had the highest results (MMSE mean 27.02). Regarding the MCI type (amnestic vs. non-amnestic), our sample was quite balanced in general terms, having percentages in the four classes ranging from approximately 51 to 66%. According to the classification of possible or probable MCI profile, the *Irritability* and *Apathy* classes had a higher percentage of patients with a diagnosis of probable amnestic (47 and 48.2%, respectively). Finally, patients in the *Irritability* and *Apathy* classes showed a higher proportion of conversion to dementia.

**TABLE 1:** Demographic characteristics and clinical variables of our final sample (*n* = 2137) stratified by neuropsychiatric symptoms cluster (NPS cluster).

	**Irritability class**	**Apathy class**	**Anxiety/Depression class**	**Asymptomatic class**	**F/χ^2^**	***p***
	***n* = 134**	***n* = 272**	***n* = 1056**	***n* = 675**		
**Age** (years)	75.17 (7.99)	76.20 (7.32)	73.82 (8.31)	75.23 (8.15)	8.30	< 0.001
**Gender** (% of females)	39 (29.1%)	112 (41.2%)	688 (65.2%)	411 (60.9%)	102.14	< 0.001
**Education** (years)	7.38 (3.80)	8.04 (4.23)	7.10 (3.79)	7.25 (4.08)	4.14	0.006
**MMSE** (total score)	27.00 (1.72)	26.65 (1.74)	27.02 (1.69)	26.97 (1.76)	3.46	0.016
**MCI type** (% of amnestic)	69 (51.5%)	180 (66.2%)	609 (57.7%)	417 (61.8%)	11.51	0.009
**MCI profile** (% of probable)	64 (47.0%)	131 (48.2%)	292 (27.7%)	266 (39.4%)	59.86	< 0.001
**Conversion to dementia**	72 (53.7%)	149 (54.8%)	394 (37.3%)	264 (39.1%)	37.21	< 0.001

*Results are shown as mean (SD) for age, education and MMSE; whereas for gender, mild cognitive impairment (MCI) type, MCI profile and conversion to dementia, data are showed as *n* (%).*

*MMSE, Mini-Mental State Examination.*

*MCI type, amnestic/non-amnestic.*

*MCI profile, probable/possible.*

*Conversion to dementia was reported independently of the etiology.*

*Class is related to neuropsychiatric-cluster belonging (Class 1 = Irritability; Class 2 = Apathy; Class 3 = Anxiety/Depression; 4 = Asymptomatic).*

**FIGURE 1:**
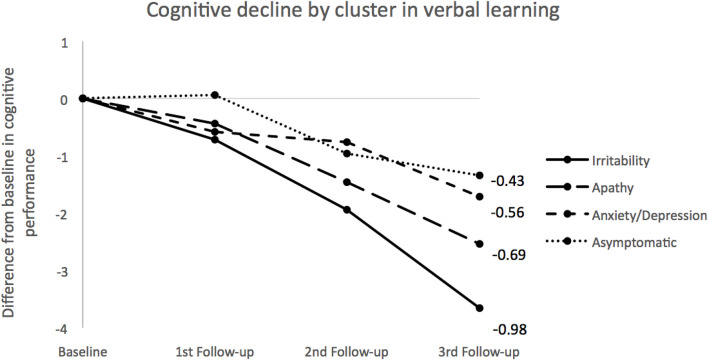
Cognitive decline across clusters for verbal learning. Measures for each group were obtained using LMM means by calculating differences between baseline and follow-ups for each time point. Slopes for each cluster were calculated using the 2-known points approach. Negative values correspond to decrements: the larger the absolute values the steeper the line. Numbers at the end of the lines indicate the cognitive slopes for each class.

**FIGURE 2:**
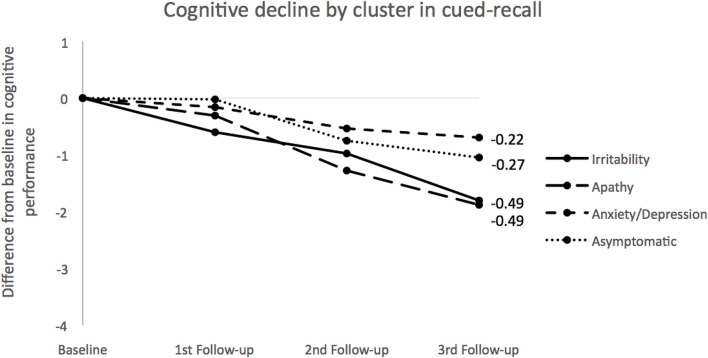
Cognitive decline across clusters for cued-recall. Measures for each group were obtained using LMM means by calculating differences between baseline and follow-ups for each time point. Slopes for each cluster were calculated using the 2-known points approach. Negative values correspond to decrements: the larger the absolute values the steeper the line. Numbers at the end of the lines indicate the cognitive slopes for each class.

Table 2 shows the LMM results for cognitive domains accounting for cluster, time, and cluster by time interaction. Only the memory domain showed a significant interaction in cluster by time, with verbal learning and cued-recall in particular being the only processes showing significant differences. Simple effects for these two cognitive functions comparing baseline with the third follow-up are displayed in Table 3, revealing significant results in all four clusters (*p* < 0.001). When differences between the final follow-up and the baseline scores were calculated in terms of verbal learning, a faster decline was shown in the *Irritability* class, with double the difference, versus a slower decline in the *Asymptomatic* class, with 3.66 and 1.34, respectively. With regard to effect size (Cohen’s D), the *Irritability* class had the highest score (0.69), whereas for *Apathy* and *Anxiety/Depression* classes the effect sizes were medium (0.49 and 0.31, respectively). The *Asymptomatic* class showed the smallest effect size (0.22). Similar results were obtained for cued-recall, but this time the *Irritability* and *Apathy* classes had quite similar differences between follow-up measures and the baseline (1.80 and 1.88, respectively), and the *Anxiety/Depression* class had the lowest score difference from the baseline. In terms of effect sizes, the *Irritability* class again showed the largest effect size (0.64), followed by the *Apathy* and *Asymptomatic* classes (0.46 and 0.30, respectively), with the *Anxiety/Depression* class having the smallest effect size (0.21). Cognitive decline for memory domains (learning and cued recall) were calculated for each cluster trajectory (see Figures 1, 2, respectively). Slopes of the trajectories were also presented for each cluster. Figures 1, 2 show speed of decline, with the *Irritability* class being the faster decliner (cognitive slope −0.98) and the *Asymptomatic* class the slower decliner (cognitive slope −0.43) in relation to verbal learning. For cued-recall, the *Irritability* and *Apathy* classes had the same cognitive slope value, and the *Anxiety/Depression* class showed a slower decline in this memory domain.

**TABLE 2:** Linear mixed model results of cluster by time interaction and main effects in cognitive domains.

	**Cluster**	**Time**	**Interaction cluster × time**
**Attention**			
Digit span forward (WAIS III)	1.59 (0.189)	12.82 (<0.001)	1.34 (0.212)
**Working memory**			
Digit span backward (WAIS III)	0.53 (0.661)	14.33 (<0.001)	1.28 (0.244)
**Processing speed**			
Execution time in sec (SKT)	6.71 (< 0.001)	1.87 (0.134)	0.59 (0.803)
**Executive**			
Phonetic fluency	6.86 (< 0.001)	5.27 (0.001)	1.59 (0.112)
Semantic fluency	9.79 (< 0.001)	55.64 (< 0.001)	0.78 (0.638)
Verbal fluency	5.40 (0.001)	0.69 (0.559)	0.73 (0.682)
Inhibition ability (SKT errors)	3.66 (0.012)	3.55 (0.014)	1.48 (0.152)
Abstract reasoning (WAIS III)	7.58 (< 0.001)	41.93 (< 0.001)	1.30 (0.230)
**Verbal memory**			
Verbal learning (WMS III)	5.27 (0.001)	45.86 (< 0.001)	**2.93 (0.002)**
Long-term retention (WMS III)	3.45 (0.016)	43.70 (< 0.001)	2.51 (0.008)
Cued-recall (WMS III)	3.15 (0.024)	45.46 (< 0.001)	**2.92 (0.002)**
**Language**			
Naming (BNT abbreviated)	0.61 (0.612)	45.05 (< 0.001)	1.10 (0.363)
**Gnosis**			
Poppelreuter	1.33 (0.262)	21.12 (< 0.001)	1.14 (0.333)
**Visuospatial skills**			
Luria’s clock	3.14 (0.024)	20.32 (< 0.001)	0.82 (0.597)
**Praxis**			
Block-design (WAIS III)	3.19 (0.023)	18.34 (< 0.001)	1.30 (0.230)
**General cognition**			
Total score (sum)	10.34 (< 0.001)	138.86 (< 0.001)	2.60 (0.006)

*Results are shown as follows: F(*p*).*

*After Bonferroni correction for multiple testing, an effect is significant when *p* < 0.003 (in bold).*

*Asymptomatic class was considered the reference category in the LMM analysis.*

*Age, MMSE, educational level, sex, conversion to dementia (yes/no), MCI type (amnestic/non-amnestic), and MCI profile (possible/probable) were also included in the models and were considered as fixed factors.*

*WAIS, Wechsler Adult Intelligence Scale; SKT, Syndrom-Kurztest; WMS, Wechsler Memory Scale; BNT, Boston Naming Test.*

*Cluster is the neuropsychiatric class (Class 1 = Irritability; Class 2 = Apathy; Class 3 = Anxiety/Depression; Class 4 = Asymptomatic).*

*Time refers to the assessment at every follow-up for our study period.*

**TABLE 3:** Simple effects and effect sizes of significant cognitive domains (i.e., verbal learning and cued-recall) between baseline (X_1_) and third follow-up (X_4_) measures.

	**Cluster**	**Δ X_1_–X_4_**	**Confidence interval for difference (95%)**	***P***	**Cohen’s *d* difference**
Verbal learning	Irritability	3.66	2.48–4.84	<0.001	0.69
	Apathy	2.54	1.59–3.48	<0.001	0.49
	Anxiety/Depression	1.72	1.24–2.19	<0.001	0.31
	Asymptomatic	1.35	0.76–1.92	<0.001	0.22
Cued recall	Irritability	1.80	1.08–2.53	<0.001	0.64
	Apathy	1.88	1.30–2.47	<0.001	0.46
	Anxiety/Depression	0.70	0.40–0.99	<0.001	0.21
	Asymptomatic	1.05	0.69–1.41	<0.001	0.30

*Cluster reflects neuropsychiatric symptoms class: Class 1 = Irritability; Class 2 = Apathy; Class 3 = Anxiety/Depression; Class 4 = Asymptomatic.*

*Confidence interval refers to mean differences.*

*Asymptomatic class was considered the reference category.*

*Age, MMSE, educational level, sex, conversion to dementia (yes/no), MCI type (amnestic/non-amnestic), and MCI profile (possible/probable) were also included in the models and were considered as fixed factors.*

*Δ X_1_–X_4_ = Difference of last follow-up from the baseline.*

## Discussion

The findings in our study revealed different trajectories of cognitive decline in memory domains depending on NPS clusters (*Irritability*, *Apathy*, *Anxiety/Depression*, and *Asymptomatic*) in patients with MCI. In our sample of 2,137 MCI patients, the *Irritability* and *Apathy* NPS classes shared a similar pattern of faster cognitive decline in two memory domains (verbal learning and cued-recall), compared to the *Anxiety/Depression* and *Asymptomatic* classes, which showed a slower cognitive worsening over the stipulated follow-up period. Even though *Irritability* was the least prevalent neuropsychiatric condition in this sample, it proved to be the NPS class with the worst and fastest cognitive decline. Therefore, the present findings suggest that although *Irritability* and *Apathy* are less frequent NPS in MCI, these symptoms should be taken into account to improve the quality and usefulness in diagnostic and prognostic evaluation of cognitive worsening in MCI patients, especially those with an amnestic profile.

Although irritability is included among the so-called affective NPS, there is no substantial literature reporting consistent results on how individuals evolve in terms of cognitive decline, neither in healthy controls (Lobo et al., 2008; Leoutsakos et al., 2015), nor in patients with MCI (Forrester et al., 2016), and/or dementia (Moran et al., 2004). Indeed, most of the studies in MCI have focused on other affective NPS such as anxiety, apathy and depression (Penna, 2013). However, some authors have postulated that irritability could be among the affective symptoms that foretell a faster decline in conversion to dementia (Ismail et al., 2017; Jang et al., 2020), but to date none have provided data on that. Therefore, the present results partially support this hypothesis, adding some novelty about which cognitive domains could be more affected, always taking into account that the resulting clusters are mainly constellations of NPS, in which one symptom is the most manifest. For instance, Irritability cluster embraced irritable symptoms (0.93), but also anxiety and apathy to a lesser extent (both 0.63). Therefore, it is possible that the differential cognitive decline observed in individuals belonging to the Irritability cluster may be somehow influenced by anxious and apathic symptoms. In any case, with the present results, this is just speculative, but future studies should explore the mechanistic process underneath the effect of NPS on cognitive decline. It can be hypothesized that the presence of irritability may confer extra vulnerability to a faster conversion to dementia. It is worth mentioning that the large sample size of this study allowed the detection of an *Irritability* class, and it is probable that previous studies failed to detect a consistent cluster comprising individuals with irritability due to the lower prevalence compared to other affective NPS.

In contrast, several studies have explored the relationship between apathy and cognitive decline. Some authors indicated an increased risk of progression from MCI to AD when apathy was presented in isolation (Vicini Chilovi et al., 2009; Richard et al., 2012), whereas others postulated the risk was even higher when combined with depressive symptoms (Ruthirakuhan et al., 2019). Strikingly, low isolated depressive symptoms were not associated with cognitive decline (Richard et al., 2012). Conversely, another recent study demonstrated that both apathy and anxiety were associated with cognitive decline when presented comorbidly (Johansson et al., 2020). Our results converged with these findings, as we observed a sharper cognitive decline suffered by patients in the *Apathy* class compared to *Anxiety/Depression*. Given that cognitive decline is one of the factors favoring conversion to dementia and it was adjusted in our analyses, the findings shed light on the NPS profiles that could entail an earlier risk of conversion, and thus act as isolated markers.

With regard to anxiety and depression, both are among the most prevalent affective NPS in MCI patients (Lyketsos et al., 2002; Zhang et al., 2012), but their influence on cognitive decline is still controversial (Chan et al., 2011). Those symptoms have mostly been considered to be precursors of dementia, whereas only a few studies considered anxiety and depression to be a mere reaction to cognitive losses perceived by the patient (Simard et al., 2009; Di Iulio et al., 2010), which could be a consequence of and not an early marker for conversion to dementia. The present findings provided evidence of no clear association between anxious and depressive symptoms and faster cognitive decline; in contrast to other studies, both symptoms did not yield a worst prognosis in our sample. Note that the cognitive trajectory of the *Anxiety/Depression* class was comparable to the *Asymptomatic* class in terms of showing no consistent cognitive decline for those two clusters, as reported by other researchers (Ismail et al., 2017; Martin and Velayudhan, 2020). These findings may suggest that although anxiety and depression are the most frequently detected and known affective NPS in patients with cognitive decline, clinicians should bear in mind other affective NPS beyond anxious and depressed manifestations that could be more relevant in the progression to dementia.

The classification of individuals by symptomatic classes rather than isolated symptoms seems to be more useful and informative as it better reflects day-to-day reality in a memory clinic. Among the different studies exploring MCI populations grouped according to comorbid NPS, significant differences exist in obtained cluster solutions, probably due to the methodological approaches used. For instance, some works used a volunteer sample (Leoutsakos et al., 2015; Forrester et al., 2016; Jang et al., 2020), whereas others used clinical samples (David et al., 2016; Siafarikas et al., 2018; Liew, 2019). The statistical approach and designs were also different among studies (i.e., LCA vs. factor analysis, techniques that group individuals vs. grouping characteristics, respectively; or cross-sectional vs. longitudinal), which could have undermined the importance of taking affective NPS into consideration in diagnostic and prognostic evaluations. One of the abovementioned publications assessed cognitive decline across latent classes (David et al., 2016). However, the authors did not include affective NPS *per se*, but rather their severity, and they only evaluated memory and executive function domains, apart from the MMSE, to obtain a global cognitive measure. Therefore, the present study represents a step forward as cognitive decline was explored in assessing different cognitive processes.

A relevant finding of the present study is therefore that not all cognitive domains were affected equally at this early stage, but instead they behaved as isolated processes that showed subtle differences in cognitive decline when NPS classes were taken into account. Likewise, the results also revealed that an accurate assessment of MCI patients, contrary to previous work in already diagnosed dementia patients (Escudero et al., 2019), should cover cognitive performance by domains as well as NPS as these can guide the prognosis of MCI, especially now, when diagnosis can be sought earlier than ever. Interestingly, different cognitive trajectories were observed according to early NPS instead of neurological symptoms, which could help clinicians be aware of a possible diagnosis of dementia or other neurodegenerative diseases (Geda et al., 2013; Dietlin et al., 2019; Wise et al., 2019), and consider what is necessary to slow down progression of the illness, where possible.

There are also limitations to be considered in the current study. First, there are baseline differences in demographic characteristics and clinical variables among NPS clusters which could undermine the findings, even though the analyses included these variables. Second, it is important to consider including medication records in future studies, as it could affect the evolution of an underlying neuropsychiatric condition. Also, a longer follow-up would be appropriate to determine how NPS and cognitive decline will interact in the long run, as well as to analyze the long-term stability of NPS classes. Also, the presence of early AD-related biomarkers would help achieve a more accurate etiological diagnosis, and to benchmark NPS observations. A third limitation is associated with the estimated variances of parameter estimates; these may have been biased because heteroscedasticity was not taken into account for the repeated measurements of individuals and consequently may have affected the precision of estimating the appropriate model. However, similar studies published so far have failed to account for heteroscedasticity and the findings are consistent. Finally, the last limitation is focused on sex perspective. In the present study it was necessary to explore over 2,100 MCI participants to generate a consistent group of 134 irritable participants. In fact, this is one of the most relevant findings of our previous study (Roberto et al., 2021). Unfortunately, in this cluster, only 39 were women. The idea to analyze data considering sex as a main factor (in interaction with cluster and time) is, of course, clinically interesting, but the distribution of this factor in the different clusters, and especially in the irritable group, prevented us to consider sex factor as a key effect. More longitudinal studies are necessary, with larger samples, to obtain consistent empirical groups of patients where cognitive trajectories in men and women could be estimated with precision.

## Conclusion

The approach of this study explores specific cognitive decline trajectories based on affective NPS clusters in MCI patients from a memory clinic, adding some novelty with respect to previous works. Specifically, and according to our results, *Irritability* and *Apathy* classes share a similar pattern of faster cognitive decline in two memory domains (verbal learning and cued-recall), compared to the *Anxiety/Depression* and *Asymptomatic* classes. The present findings emphasize the relevance of including an assessment of affective NPS when starting a diagnostic process provided that such symptoms—and in particular irritability and apathy—might act as aggravating factors. Our findings appear to open a new avenue to use NPS assessment as a clinical tool of great value when it comes to detecting in advance which patients could suffer from a marked worsening in cognition.

## Data Availability Statement

The data that support this study may be provided upon reasonable request. Requests to access the datasets should be directed to SV, svalero@fundacioace.org.

## Ethics Statement

The studies involving human participants were reviewed and approved by Hospital Clínic i Provincial of Barcelona, Spain. The patients/participants provided their written informed consent to participate in this study.

## Author Contributions

NR, MP, LT, MB, and SV conceived the idea, designed the study, and wrote the protocol and methodology. MM, MA, IH, AM, MR-R, CA, EE, SM-G, JT, LV, AE, GO, AP-C, ÁS, AO, IR, SM-G, and LM acquired all the data, performed technical procedures, and managed the data set. NR, MM, and AR managed previous literature searches. NR, MP, and SV contributed to the statistical analysis, interpretation of the results and writing of the first draft of the manuscript. EA-M helped in the statistical analysis and interpretation of the results. MM, MA, AR, LT, and MB revised the manuscript critically for intellectual content. All authors contributed to the writing of the final version and approved the manuscript.

## Conflict of Interest

The authors declare that the research was conducted in the absence of any commercial or financial relationships that could be construed as a potential conflict of interest.

## Publisher’s Note

All claims expressed in this article are solely those of the authors and do not necessarily represent those of their affiliated organizations, or those of the publisher, the editors and the reviewers. Any product that may be evaluated in this article, or claim that may be made by its manufacturer, is not guaranteed or endorsed by the publisher.
