# Supplementary material for: Neuropsychiatric Profile as a Predictor of Cognitive Decline in Mild Cognitive Impairment
Source: Front Aging Neurosci. 2021 Dec 8;13:718949. doi: 10.3389/fnagi.2021.718949 (PMC8693625; doi:10.3389/fnagi.2021.718949)
Supplement: Supplementary file 1 [file Data_Sheet_1.docx]

********

FFEE

MIXED FE_anflu BY sex conversio_validada_febrer19 cluster_rr Índice1 amnestic_noamnestic DCL_prob_poss WITH MMSE_DN.1 edat

Anys_Escolaritat_imputada

/CRITERIA=CIN(95) MXITER(200) MXSTEP(10) SCORING(1) SINGULAR(0.000000000001) HCONVERGE(0,

ABSOLUTE) LCONVERGE(0, ABSOLUTE) PCONVERGE(0.000001, ABSOLUTE)

/FIXED= sex conversio_validada_febrer19 cluster_rr Índice1 amnestic_noamnestic DCL_prob_poss MMSE_DN.1 edat Anys_Escolaritat_imputada

cluster_rr*Índice1 | SSTYPE(3)

/METHOD=REML

/PRINT=DESCRIPTIVES SOLUTION

/RANDOM=INTERCEPT cluster_rr Índice1 cluster_rr*Índice1 | SUBJECT(Interno) COVTYPE(VC)

/REPEATED=Índice1 | SUBJECT(Interno) COVTYPE(DIAG)

/EMMEANS=TABLES(cluster_rr*Índice1) COMPARE(cluster_rr) ADJ(LSD)

/EMMEANS=TABLES(cluster_rr*Índice1) COMPARE(índice1) ADJ(LSD).

MIXED FE_Pflu BY sex conversio_validada_febrer19 cluster_rr Índice1 amnestic_noamnestic DCL_prob_poss WITH MMSE_DN.1 edat

Anys_Escolaritat_imputada

/CRITERIA=CIN(95) MXITER(200) MXSTEP(10) SCORING(1) SINGULAR(0.000000000001) HCONVERGE(0,

ABSOLUTE) LCONVERGE(0, ABSOLUTE) PCONVERGE(0.000001, ABSOLUTE)

/FIXED= sex conversio_validada_febrer19 cluster_rr Índice1 amnestic_noamnestic DCL_prob_poss MMSE_DN.1 edat Anys_Escolaritat_imputada

cluster_rr*Índice1 | SSTYPE(3)

/METHOD=REML

/PRINT=DESCRIPTIVES SOLUTION

/RANDOM=INTERCEPT cluster_rr Índice1 cluster_rr*Índice1 | SUBJECT(Interno) COVTYPE(VC)

/REPEATED=Índice1 | SUBJECT(Interno) COVTYPE(DIAG)

/EMMEANS=TABLES(cluster_rr*Índice1) COMPARE(cluster_rr) ADJ(LSD)

/EMMEANS=TABLES(cluster_rr*Índice1) COMPARE(índice1) ADJ(LSD).

MIXED FE_R_abstracte BY sex conversio_validada_febrer19 cluster_rr Índice1 amnestic_noamnestic DCL_prob_poss WITH MMSE_DN.1 edat

Anys_Escolaritat_imputada

/CRITERIA=CIN(95) MXITER(200) MXSTEP(10) SCORING(1) SINGULAR(0.000000000001) HCONVERGE(0,

ABSOLUTE) LCONVERGE(0, ABSOLUTE) PCONVERGE(0.000001, ABSOLUTE)

/FIXED= sex conversio_validada_febrer19 cluster_rr Índice1 amnestic_noamnestic DCL_prob_poss MMSE_DN.1 edat Anys_Escolaritat_imputada

cluster_rr*Índice1 | SSTYPE(3)

/METHOD=REML

/PRINT=DESCRIPTIVES SOLUTION

/RANDOM=INTERCEPT cluster_rr Índice1 cluster_rr*Índice1 | SUBJECT(Interno) COVTYPE(VC)

/REPEATED=Índice1 | SUBJECT(Interno) COVTYPE(DIAG)

/EMMEANS=TABLES(cluster_rr*Índice1) COMPARE(cluster_rr) ADJ(LSD)

/EMMEANS=TABLES(cluster_rr*Índice1) COMPARE(índice1) ADJ(LSD).

MIXED FE_SKTerrors BY sex conversio_validada_febrer19 cluster_rr Índice1 amnestic_noamnestic DCL_prob_poss WITH MMSE_DN.1 edat

Anys_Escolaritat_imputada

/CRITERIA=CIN(95) MXITER(200) MXSTEP(10) SCORING(1) SINGULAR(0.000000000001) HCONVERGE(0,

ABSOLUTE) LCONVERGE(0, ABSOLUTE) PCONVERGE(0.000001, ABSOLUTE)

/FIXED= sex conversio_validada_febrer19 cluster_rr Índice1 amnestic_noamnestic DCL_prob_poss MMSE_DN.1 edat Anys_Escolaritat_imputada

cluster_rr*Índice1 | SSTYPE(3)

/METHOD=REML

/PRINT=DESCRIPTIVES SOLUTION

/RANDOM=INTERCEPT cluster_rr Índice1 cluster_rr*Índice1 | SUBJECT(Interno) COVTYPE(VC)

/REPEATED=Índice1 | SUBJECT(Interno) COVTYPE(DIAG)

/EMMEANS=TABLES(cluster_rr*Índice1) COMPARE(cluster_rr) ADJ(LSD)

/EMMEANS=TABLES(cluster_rr*Índice1) COMPARE(índice1) ADJ(LSD).

MIXED FE_SKTtemps BY sex conversio_validada_febrer19 cluster_rr Índice1 amnestic_noamnestic DCL_prob_poss WITH MMSE_DN.1 edat

Anys_Escolaritat_imputada

/CRITERIA=CIN(95) MXITER(200) MXSTEP(10) SCORING(1) SINGULAR(0.000000000001) HCONVERGE(0,

ABSOLUTE) LCONVERGE(0, ABSOLUTE) PCONVERGE(0.000001, ABSOLUTE)

/FIXED= sex conversio_validada_febrer19 cluster_rr Índice1 amnestic_noamnestic DCL_prob_poss MMSE_DN.1 edat Anys_Escolaritat_imputada

cluster_rr*Índice1 | SSTYPE(3)

/METHOD=REML

/PRINT=DESCRIPTIVES SOLUTION

/RANDOM=INTERCEPT cluster_rr Índice1 cluster_rr*Índice1 | SUBJECT(Interno) COVTYPE(VC)

/REPEATED=Índice1 | SUBJECT(Interno) COVTYPE(DIAG)

/EMMEANS=TABLES(cluster_rr*Índice1) COMPARE(cluster_rr) ADJ(LSD)

/EMMEANS=TABLES(cluster_rr*Índice1) COMPARE(índice1) ADJ(LSD).

MIXED Fluidesa_Verbal_Accio BY sex conversio_validada_febrer19 cluster_rr Índice1 amnestic_noamnestic DCL_prob_poss WITH MMSE_DN.1 edat

Anys_Escolaritat_imputada

/CRITERIA=CIN(95) MXITER(200) MXSTEP(10) SCORING(1) SINGULAR(0.000000000001) HCONVERGE(0,

ABSOLUTE) LCONVERGE(0, ABSOLUTE) PCONVERGE(0.000001, ABSOLUTE)

/FIXED= sex conversio_validada_febrer19 cluster_rr Índice1 amnestic_noamnestic DCL_prob_poss MMSE_DN.1 edat Anys_Escolaritat_imputada

cluster_rr*Índice1 | SSTYPE(3)

/METHOD=REML

/PRINT=DESCRIPTIVES SOLUTION

/RANDOM=INTERCEPT cluster_rr Índice1 cluster_rr*Índice1 | SUBJECT(Interno) COVTYPE(VC)

/REPEATED=Índice1 | SUBJECT(Interno) COVTYPE(DIAG)

/EMMEANS=TABLES(cluster_rr*Índice1) COMPARE(cluster_rr) ADJ(LSD)

/EMMEANS=TABLES(cluster_rr*Índice1) COMPARE(índice1) ADJ(LSD).

Att&WM

MIXED M_digttotal_direct BY sex conversio_validada_febrer19 cluster_rr Índice1 amnestic_noamnestic DCL_prob_poss WITH MMSE_DN.1 edat

Anys_Escolaritat_imputada

/CRITERIA=CIN(95) MXITER(200) MXSTEP(10) SCORING(1) SINGULAR(0.000000000001) HCONVERGE(0,

ABSOLUTE) LCONVERGE(0, ABSOLUTE) PCONVERGE(0.000001, ABSOLUTE)

/FIXED= sex conversio_validada_febrer19 cluster_rr Índice1 amnestic_noamnestic DCL_prob_poss MMSE_DN.1 edat Anys_Escolaritat_imputada

cluster_rr*Índice1 | SSTYPE(3)

/METHOD=REML

/PRINT=DESCRIPTIVES SOLUTION

/RANDOM=INTERCEPT cluster_rr Índice1 cluster_rr*Índice1 | SUBJECT(Interno) COVTYPE(VC)

/REPEATED=Índice1 | SUBJECT(Interno) COVTYPE(DIAG)

/EMMEANS=TABLES(cluster_rr*Índice1) COMPARE(cluster_rr) ADJ(LSD)

/EMMEANS=TABLES(cluster_rr*Índice1) COMPARE(índice1) ADJ(LSD).

MIXED M_digttotal_invers BY sex conversio_validada_febrer19 cluster_rr Índice1 amnestic_noamnestic DCL_prob_poss WITH MMSE_DN.1 edat

Anys_Escolaritat_imputada

/CRITERIA=CIN(95) MXITER(200) MXSTEP(10) SCORING(1) SINGULAR(0.000000000001) HCONVERGE(0,

ABSOLUTE) LCONVERGE(0, ABSOLUTE) PCONVERGE(0.000001, ABSOLUTE)

/FIXED= sex conversio_validada_febrer19 cluster_rr Índice1 amnestic_noamnestic DCL_prob_poss MMSE_DN.1 edat Anys_Escolaritat_imputada

cluster_rr*Índice1 | SSTYPE(3)

/METHOD=REML

/PRINT=DESCRIPTIVES SOLUTION

/RANDOM=INTERCEPT cluster_rr Índice1 cluster_rr*Índice1 | SUBJECT(Interno) COVTYPE(VC)

/REPEATED=Índice1 | SUBJECT(Interno) COVTYPE(DIAG)

/EMMEANS=TABLES(cluster_rr*Índice1) COMPARE(cluster_rr) ADJ(LSD)

/EMMEANS=TABLES(cluster_rr*Índice1) COMPARE(índice1) ADJ(LSD).

Mem verbal

MIXED M_recon BY sex conversio_validada_febrer19 cluster_rr Índice1 amnestic_noamnestic DCL_prob_poss WITH MMSE_DN.1 edat

Anys_Escolaritat_imputada

/CRITERIA=CIN(95) MXITER(200) MXSTEP(10) SCORING(1) SINGULAR(0.000000000001) HCONVERGE(0,

ABSOLUTE) LCONVERGE(0, ABSOLUTE) PCONVERGE(0.000001, ABSOLUTE)

/FIXED= sex conversio_validada_febrer19 cluster_rr Índice1 amnestic_noamnestic DCL_prob_poss MMSE_DN.1 edat Anys_Escolaritat_imputada

cluster_rr*Índice1 | SSTYPE(3)

/METHOD=REML

/PRINT=DESCRIPTIVES SOLUTION

/RANDOM=INTERCEPT cluster_rr Índice1 cluster_rr*Índice1 | SUBJECT(Interno) COVTYPE(VC)

/REPEATED=Índice1 | SUBJECT(Interno) COVTYPE(DIAG)

/EMMEANS=TABLES(cluster_rr*Índice1) COMPARE(cluster_rr) ADJ(LSD)

/EMMEANS=TABLES(cluster_rr*Índice1) COMPARE(índice1) ADJ(LSD).

MIXED M_ret BY sex conversio_validada_febrer19 cluster_rr Índice1 amnestic_noamnestic DCL_prob_poss WITH MMSE_DN.1 edat

Anys_Escolaritat_imputada

/CRITERIA=CIN(95) MXITER(200) MXSTEP(10) SCORING(1) SINGULAR(0.000000000001) HCONVERGE(0,

ABSOLUTE) LCONVERGE(0, ABSOLUTE) PCONVERGE(0.000001, ABSOLUTE)

/FIXED= sex conversio_validada_febrer19 cluster_rr Índice1 amnestic_noamnestic DCL_prob_poss MMSE_DN.1 edat Anys_Escolaritat_imputada

cluster_rr*Índice1 | SSTYPE(3)

/METHOD=REML

/PRINT=DESCRIPTIVES SOLUTION

/RANDOM=INTERCEPT cluster_rr Índice1 cluster_rr*Índice1 | SUBJECT(Interno) COVTYPE(VC)

/REPEATED=Índice1 | SUBJECT(Interno) COVTYPE(DIAG)

/EMMEANS=TABLES(cluster_rr*Índice1) COMPARE(cluster_rr) ADJ(LSD)

/EMMEANS=TABLES(cluster_rr*Índice1) COMPARE(índice1) ADJ(LSD).

MIXED M_wms_total BY sex conversio_validada_febrer19 cluster_rr Índice1 amnestic_noamnestic DCL_prob_poss WITH MMSE_DN.1 edat

Anys_Escolaritat_imputada

/CRITERIA=CIN(95) MXITER(200) MXSTEP(10) SCORING(1) SINGULAR(0.000000000001) HCONVERGE(0,

ABSOLUTE) LCONVERGE(0, ABSOLUTE) PCONVERGE(0.000001, ABSOLUTE)

/FIXED= sex conversio_validada_febrer19 cluster_rr Índice1 amnestic_noamnestic DCL_prob_poss MMSE_DN.1 edat Anys_Escolaritat_imputada

cluster_rr*Índice1 | SSTYPE(3)

/METHOD=REML

/PRINT=DESCRIPTIVES SOLUTION

/RANDOM=INTERCEPT cluster_rr Índice1 cluster_rr*Índice1 | SUBJECT(Interno) COVTYPE(VC)

/REPEATED=Índice1 | SUBJECT(Interno) COVTYPE(DIAG)

/EMMEANS=TABLES(cluster_rr*Índice1) COMPARE(cluster_rr) ADJ(LSD)

/EMMEANS=TABLES(cluster_rr*Índice1) COMPARE(índice1) ADJ(LSD).

Leng

MIXED LL_Namingtotal BY sex conversio_validada_febrer19 cluster_rr Índice1 amnestic_noamnestic DCL_prob_poss WITH MMSE_DN.1 edat

Anys_Escolaritat_imputada

/CRITERIA=CIN(95) MXITER(200) MXSTEP(10) SCORING(1) SINGULAR(0.000000000001) HCONVERGE(0,

ABSOLUTE) LCONVERGE(0, ABSOLUTE) PCONVERGE(0.000001, ABSOLUTE)

/FIXED= sex conversio_validada_febrer19 cluster_rr Índice1 amnestic_noamnestic DCL_prob_poss MMSE_DN.1 edat Anys_Escolaritat_imputada

cluster_rr*Índice1 | SSTYPE(3)

/METHOD=REML

/PRINT=DESCRIPTIVES SOLUTION

/RANDOM=INTERCEPT cluster_rr Índice1 cluster_rr*Índice1 | SUBJECT(Interno) COVTYPE(VC)

/REPEATED=Índice1 | SUBJECT(Interno) COVTYPE(DIAG)

/EMMEANS=TABLES(cluster_rr*Índice1) COMPARE(cluster_rr) ADJ(LSD)

/EMMEANS=TABLES(cluster_rr*Índice1) COMPARE(índice1) ADJ(LSD).

Gnosias

MIXED G_Luria BY sex conversio_validada_febrer19 cluster_rr Índice1 amnestic_noamnestic DCL_prob_poss WITH MMSE_DN.1 edat

Anys_Escolaritat_imputada

/CRITERIA=CIN(95) MXITER(200) MXSTEP(10) SCORING(1) SINGULAR(0.000000000001) HCONVERGE(0,

ABSOLUTE) LCONVERGE(0, ABSOLUTE) PCONVERGE(0.000001, ABSOLUTE)

/FIXED= sex conversio_validada_febrer19 cluster_rr Índice1 amnestic_noamnestic DCL_prob_poss MMSE_DN.1 edat Anys_Escolaritat_imputada

cluster_rr*Índice1 | SSTYPE(3)

/METHOD=REML

/PRINT=DESCRIPTIVES SOLUTION

/RANDOM=INTERCEPT cluster_rr Índice1 cluster_rr*Índice1 | SUBJECT(Interno) COVTYPE(VC)

/REPEATED=Índice1 | SUBJECT(Interno) COVTYPE(DIAG)

/EMMEANS=TABLES(cluster_rr*Índice1) COMPARE(cluster_rr) ADJ(LSD)

/EMMEANS=TABLES(cluster_rr*Índice1) COMPARE(índice1) ADJ(LSD).

MIXED G_pop_total BY sex conversio_validada_febrer19 cluster_rr Índice1 amnestic_noamnestic DCL_prob_poss WITH MMSE_DN.1 edat

Anys_Escolaritat_imputada

/CRITERIA=CIN(95) MXITER(200) MXSTEP(10) SCORING(1) SINGULAR(0.000000000001) HCONVERGE(0,

ABSOLUTE) LCONVERGE(0, ABSOLUTE) PCONVERGE(0.000001, ABSOLUTE)

/FIXED= sex conversio_validada_febrer19 cluster_rr Índice1 amnestic_noamnestic DCL_prob_poss MMSE_DN.1 edat Anys_Escolaritat_imputada

cluster_rr*Índice1 | SSTYPE(3)

/METHOD=REML

/PRINT=DESCRIPTIVES SOLUTION

/RANDOM=INTERCEPT cluster_rr Índice1 cluster_rr*Índice1 | SUBJECT(Interno) COVTYPE(VC)

/REPEATED=Índice1 | SUBJECT(Interno) COVTYPE(DIAG)

/EMMEANS=TABLES(cluster_rr*Índice1) COMPARE(cluster_rr) ADJ(LSD)

/EMMEANS=TABLES(cluster_rr*Índice1) COMPARE(índice1) ADJ(LSD).

Praxias

MIXED P_constr_total BY sex conversio_validada_febrer19 cluster_rr Índice1 amnestic_noamnestic DCL_prob_poss WITH MMSE_DN.1 edat

Anys_Escolaritat_imputada

/CRITERIA=CIN(95) MXITER(200) MXSTEP(10) SCORING(1) SINGULAR(0.000000000001) HCONVERGE(0,

ABSOLUTE) LCONVERGE(0, ABSOLUTE) PCONVERGE(0.000001, ABSOLUTE)

/FIXED= sex conversio_validada_febrer19 cluster_rr Índice1 amnestic_noamnestic DCL_prob_poss MMSE_DN.1 edat Anys_Escolaritat_imputada

cluster_rr*Índice1 | SSTYPE(3)

/METHOD=REML

/PRINT=DESCRIPTIVES SOLUTION

/RANDOM=INTERCEPT cluster_rr Índice1 cluster_rr*Índice1 | SUBJECT(Interno) COVTYPE(VC)

/REPEATED=Índice1 | SUBJECT(Interno) COVTYPE(DIAG)

/EMMEANS=TABLES(cluster_rr*Índice1) COMPARE(cluster_rr) ADJ(LSD)

/EMMEANS=TABLES(cluster_rr*Índice1) COMPARE(índice1) ADJ(LSD).

NPS total

MIXED total_np BY sex conversio_validada_febrer19 cluster_rr Índice1 amnestic_noamnestic DCL_prob_poss WITH MMSE_DN.1 edat

Anys_Escolaritat_imputada

/CRITERIA=CIN(95) MXITER(200) MXSTEP(10) SCORING(1) SINGULAR(0.000000000001) HCONVERGE(0,

ABSOLUTE) LCONVERGE(0, ABSOLUTE) PCONVERGE(0.000001, ABSOLUTE)

/FIXED= sex conversio_validada_febrer19 cluster_rr Índice1 amnestic_noamnestic DCL_prob_poss MMSE_DN.1 edat Anys_Escolaritat_imputada

cluster_rr*Índice1 | SSTYPE(3)

/METHOD=REML

/PRINT=DESCRIPTIVES SOLUTION

/RANDOM=INTERCEPT cluster_rr Índice1 cluster_rr*Índice1 | SUBJECT(Interno) COVTYPE(VC)

/REPEATED=Índice1 | SUBJECT(Interno) COVTYPE(DIAG)

/EMMEANS=TABLES(cluster_rr*Índice1) COMPARE(cluster_rr) ADJ(LSD)

/EMMEANS=TABLES(cluster_rr*Índice1) COMPARE(índice1) ADJ(LSD).
